# Enhanced metastasis in RNF13 knockout mice is mediated by a reduction in GM-CSF levels

**DOI:** 10.1007/s13238-015-0188-7

**Published:** 2015-07-22

**Authors:** He Cheng, Aodi Wang, Jiao Meng, Yong Zhang, Dahai Zhu

**Affiliations:** The National Laboratory of Medical Molecular Biology, Institute of Basic Medical Sciences, Chinese Academy of Medical Sciences and School of Basic Medicine, Peking Union Medical College, Beijing, 100005 China

**Keywords:** RNF13, metastasis, GM-CSF, lung cancer

## Abstract

RING finger protein 13 (RNF13) is a novel E3 ubiquitin ligase whose expression is associated with cancer development. However, its specific role in cancer progression and metastasis remains unclear. Here, a B16F10/LLC experimental pulmonary metastatic model was developed to examine the formation of metastatic foci in the lung. A greater number of tumor colonies were observed in the lungs of RNF13-knockout (KO) mice than in their wild-type (WT) littermates, whereas no significant differences in tumor size were observed between the two groups. In short-term experiments, the number of fluorescently-labeled B16F10 cells increased remarkably in RNF13-KO lungs at early time points, whereas clearance of tumor cells from the blood was not affected. These results indicated that RNF13 may inhibit the colonization of B16F10 cells in the lung. Assessment of the concentration of various cytokines in tumor bearing lungs and blood did not detect significant differences between the blood of RNF13-KO and WT mice; however the levels of GM-CSF were significantly reduced in RNF13-KO tumor bearing lungs, which may have guided more B16F10 cells to migrate to the lungs. This was confirmed by lower GM-CSF concentrations in conditioned media from the culture of RNF13-KO lung slices. Collectively, our results suggest that host RNF13 affects the concentration of GM-CSF in tumor-bearing lungs, leading to a reduction in the colonization of metastatic tumor cells in the lung.

**Electronic supplementary material:**

The online version of this article (doi:10.1007/s13238-015-0188-7) contains supplementary material, which is available to authorized users.

## INTRODUCTION

Metastasis is the main cause of cancer-related death and it occurs through a cascade of events (Talmadge and Fidler, 2010). The metastatic process begins with the local invasion of malignant cells, followed by intravasation of these cells into nearby blood and lymphatic vessels, transit through the vascular system, extravasation to target organs, and the formation of micrometastases. Finally, these micrometastatic lesions grow into macroscopic tumors. In this process, tumor cells from a primary tumor successfully invade and colonize a distant organ (Hanahan and Weinberg, 2011). Despite the fact that millions of tumor cells are shed into the vasculature, few metastatic tumor cells survive and form secondary tumors; however, a similar metastatic process results in different outcomes among patients (Luzzi et al., 1998). In recent decades, to explain these discrepancies, researchers have focused mainly on the differences between cell-autonomous phenomena in malignant tumors. However, during the multistep process of tumor metastasis, the host microenvironment, which contains non-cancerous host factors, plays an active and indispensable role. One possible explanation was that the host microenvironment may influence the metastatic efficiency of tumor cells, resulting in a small subpopulation of cells capable of completing the steps required to successfully colonize a distant site, while differences in host factors among individuals may lead to the different outcomes of patients (Irmisch and Huelsken, 2013; Nguyen et al., 2009). As early as 1889, Stephen Paget proposed the “seed and soil” theory of metastasis, which suggested that specific organ microenvironments (the ‘soil’) could influence the growth of tumor cells (the ‘seeds’) (Paget, 1989). The important role of the host microenvironment in the invasion and metastasis of tumor cells is widely accepted. The interaction between tumor cells and the host microenvironment, which is referred to as “the tumor microenvironment”, determines the final fate of these metastatic tumor cells (Spano and Zollo, 2012).

RING finger protein 13 (RNF13) is an E3 ubiquitin ligase that was first identified as a gene whose expression changes in chicken embryo brain (CEB) cells in response to binding to the extracellular matrix molecule cytotactin/tenascin (CT/TN) (Tranque et al., 1996). It was later found to be ubiquitously expressed in various chicken, mouse, and human tissues (Bocock et al., 2009; Zhang et al., 2009; Zhang et al., 2010). RNF13 is involved in many physiological processes. Our previous work showed that deletion of RNF13 in mice leads to a significant deficit in spatial learning and accelerates muscle regeneration, which is mediated by macrophage-secreted cytokines (Meng et al., 2014; Zhang et al., 2013). Emerging evidence suggests that RNF13 functions in cancer development. Our previous research in pancreatic ductal adenocarcinoma showed that RNF13 expression is significantly associated with histological grading (Zhang et al., 2009). Human tissue microarray analysis also suggested an association between RNF13 expression and human colon cancer development, as the RNF13 protein is expressed at higher levels in human colon cancer samples than in control samples. Microarray analysis of multiple tumor samples suggested a link between RNF13 expression and various human tumors including melanoma (Jin et al., 2011). Van Dijk et al. (2014) identified loss-of-function mutations of RNF13 in tumor tissues isolated from several cancer patients with different tumor types, including lung and skin cancer. These data indicated that RNF13 may function during cancer development.

Nonetheless, many aspects of the molecular mechanisms that link host gene function, the tumor microenvironment, and the establishment of tumor colonies in a distant organ remain unresolved. In this regard, experimental animal models may provide valuable insight into the molecular mechanisms linking host gene expression and pulmonary colonization of tumor cells. In the present study, we established a B16F10 experimental metastatic model in RNF13-knockout (KO) mice and tracked the changes of tumor cells during this experimental metastasis. Our results indicated that the host RNF13 could inhibit experimental lung metastasis. The absence of host RNF13 lowered the concentration of GM-CSF in tumor bearing lungs, leading to enhanced invasion of melanoma cells and increased lung colonization. These data suggest that RNF13 plays a regulatory role in this process.

## RESULTS

### Elevated levels of metastasis in the lungs of mice lacking RNF13

**Figure 1:**
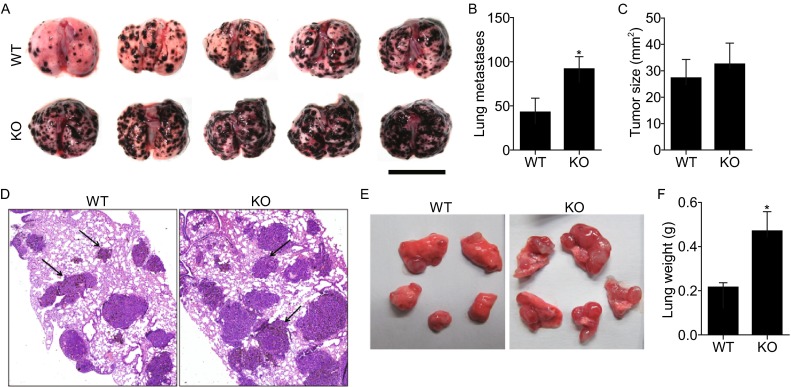
**Enhanced experimental pulmonary metastasis in host mice with RNF13 gene deletion**. (A) Representative image of pulmonary metastatic foci produced 14 days after intravenous injection of B16F10 cells. Scale bar = 1 cm. (B) Numbers of experimental pulmonary B16F10 metastases in the whole lung (*, *P* = 0.0434. *n* = 8). (C) Size distributions of B16F10 metastatic foci (ns, *P* = 0.6369. *n* = 8) (D) Representative H&E staining of sections of lungs from wild type and RNF13-KO mice treated as in (A). Arrows point to metastatic foci. Magnification 40×. (E) Representative image of pulmonary metastatic foci produced 21 days after intravenous injection of LLC cells. (F) Tumor-bearing lung weights of RNF13-KO and wild type mice 21 days after bearing LLC tumors (*, *P* = 0.0178. *n* = 6)

Our previous work suggested a link between RNF13 and tumor progression. A study that included 72 pancreatic ductal adenocarcinoma (PDAC) patients showed that RNF13 was overexpressed in 30 tumor samples, and its expression was significantly associated with histological grading (Zhang et al., 2009). In the present study, RNF13 was regarded as a host factor. To determine whether host RNF13 could influence cancer progression, in particular cancer metastasis, RNF13-KO mice were used to establish a pulmonary metastatic model. B16F10 melanoma cells were injected into the dorsal tail vein of the RNF13-KO mice and their WT littermates, which resulted in increased metastasis in RNF13-KO mice bearing B16F10 cells for 14 days (Fig. 1A). The number of metastatic foci on the lung surface of RNF13 KO mice was higher than that of their WT littermates (Fig. 1B). No significant differences in lung tumor size were observed between RNF13-KO and WT mice (Fig. 1C), and these results were confirmed by histological analysis (Fig. 1D). In the LLC experimental metastasis model, equal numbers of LLC cells were injected by tail vein, and metastasis was analyzed after 21 days. RNF13-KO mice showed a larger metastatic area in the lung and a heavier tumor-bearing lung tissue than their WT littermates (Fig. 1E and 1F). Pathological sections showed that tumor number was increased in RNF13-KO mice (Fig. S1). In mice bearing LLC cells for 27 days, the eight RNF13-KO mice developed lung cancer. Among these RNF13-KO mice, 1/8 died from the tumor metastasis, and 1/8 developed pulmonary, diaphragmatic, and mesenteric metastasis, while no tumors were detected in other tissues besides the lung in six WT mice. In these B16F10 metastatic models, RNF13 deficiency had no effect on the distribution of individual metastatic foci, suggesting that local growth was unperturbed. To further assess local growth effects, LLC cells were injected subcutaneous into the dorsal flank of RNF13-KO and WT mice. The real-time size of tumors was measured every two days and tumor weight was measured at 22 days after injection (Fig. S2A and S2B). The results showed no significant differences in tumor size and tumor weight between RNF13-KO mice and their WT littermates.

### Host RNF13 affects B16F10 cell colonization in the lung during the early stages of the experimental metastasis

**Figure 2:**
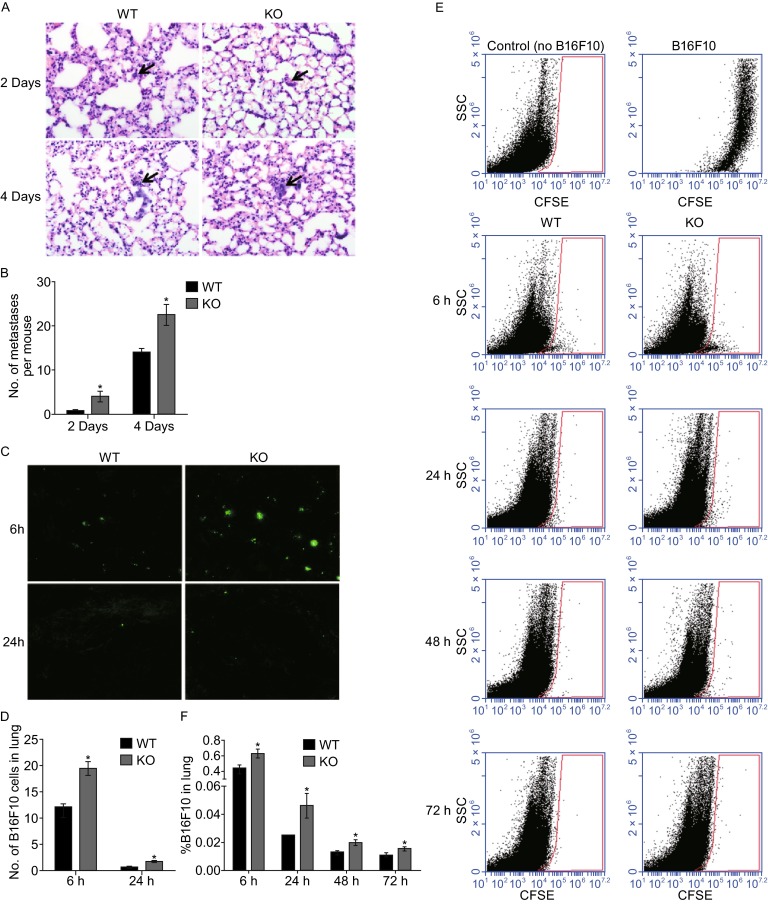
**Host RNF13 affects lung colonization**. (A) H&E staining of lung tissue sections from RNF13-KO and wild type mice at 2 days and 4 days after injection of B16F10 cells. Arrows point to micrometastases. Magnification: 400×. (B) The number of micrometastases per mouse in the same sections as in (A), (*, 2 days *P* = 0.0407. *n* = 5, 4 days *P* = 0.0161. *n* = 5). B16F10 cells labeled with BCECF or CFSE were injected into mouse tail veins. (C) Fluorescent B16F10 cells (BCECF-positive) in cryosections at 6 h and 24 h after injection. (D) Quantification of fluorescent B16F10 cells in all five lung lobes in cryosections (*, 6 h *P* = 0.0260. *n* = 6, 24 h *P* = 0.0121. *n* = 6). (E) CFSE labeled cells (relative to total suspended lung cells) were also determined using flow cytometry at 6–72 h after injection. (F) Quantification of labeled cells in (E) by flow cytometry (*, 6 h *P* = 0.0242. *n* = 6, 24 h *P* = 0.0481. *n* = 6, 48 h *P* = 0.0472. *n* = 5, 72 h *P* = 0.0420. *n* = 5)

In our tumor models, host RNF13 deficiency had no effect on tumor growth, whereas it caused an appreciable increase in metastasis. To examine the mechanism of increased metastasis in RNF13-KO mice, the distribution of B16F10 cells and metastatic clones were examined in the lung at early time points after cell injection. Equal numbers of B16F10 cells were injected through the tail vein into RNF13-KO mice and their WT littermates. At 2 and 4 days after injection, mice were sacrificed and B16F10 bearing lungs were collected. Since there were no visible metastases on the surface of the lungs at these two time points, lung tissues were fixed and sectioned for H&E staining. Histological analysis showed few single B16F10 cells in lung tissues at 2 days; micrometastases composed of several tumor cells appeared in the lungs at 4 days (Fig. 2A). Data quantification indicated that the number of either single tumor cells (2 days) or micrometastases (4 days) was higher in RNF13-KO mice than in their WT littermates (Fig. 2B). To determine whether RNF13 functions at an earlier time point in this experimental model, BCECF-labeled B16F10 cells were injected into the tail vein of mice and lungs were isolated at 6 h and 24 h. RNF13-KO lungs contained significantly more B16F10 cells than WT mice, as shown in cryosections (Fig. 2C and 2D). For 6–72 h analyses, CFSE labeled B16F10 cells were injected into the tail vein and the isolated perfused lungs were digested to prepare single cell suspensions. The relative percentage of CFSE-labeled B16F10 cells was determined by flow cytometry (Fig. 2E). A significantly greater number of B16F10 cells were recovered from RNF13-KO mice than from WT mice at 6 h, 24 h, 48 h, and 72 h (Fig. 2F). These results indicated that host RNF13 may influence the colonization of metastatic tumor cells in the lung.

### The deficiency of host RNF13 had no effect on the clearance of B16F10 cells from the blood

**Figure 3:**
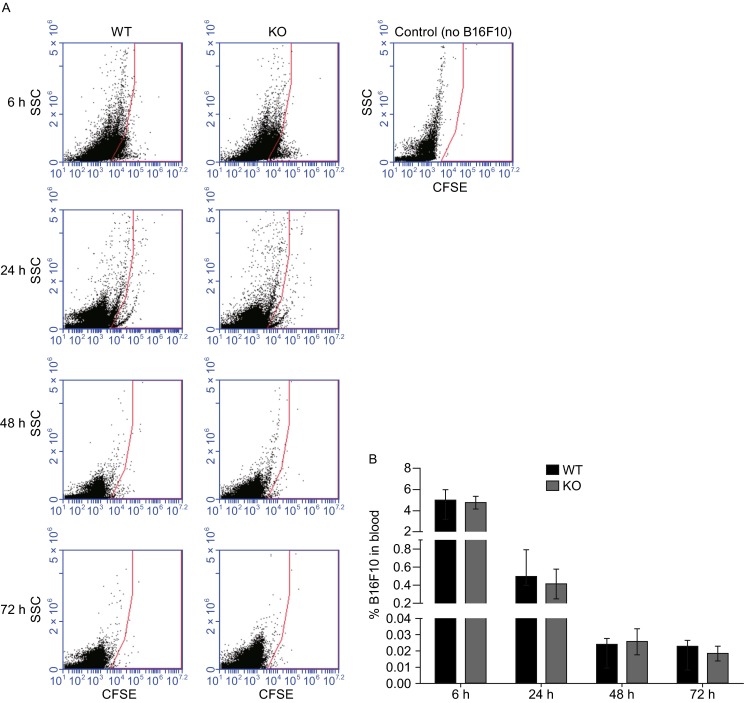
**B16F10 cells in blood**. B16F10 cells labeled with CFSE were injected into mouse tail veins. (A) After 6–72 h, blood samples were collected directly from the heart and the percentage of CFSE-labeled cells was determined by the flow cytometry (SSC = side scatter). Note that blood from a control mouse (no B16F10 injection) contained essentially no CFSE-labeled cells. (B) Quantification of labeled cells by flow cytometry (ns, 6 h *P* = 0.6831. *n* = 5, 24 h *P* = 0.8159. *n* = 6, 48 h *P* = 0.8221. *n* = 5, 72 h *P* = 0.4778. *n* = 5)

In our B16F10 experimental pulmonary metastatic model, tumor cells were injected into the circulation of mice and allowed to travel in the vascular system. Differences in the rapid clearance of cells from the blood may reflect the number of B16F10 cells available for lung colonization in RNF13-KO mice. To determine whether rapid clearance from blood was affected in RNF13-KO mice, CFSE labeled B16F10 cells were injected into the tail vein of mice, and fluorescent cells were detected by flow cytometry in the lungs and blood. Consistent with our previous results, RNF13-KO mice had a greater number of fluorescent B16F10 cells in the lung than WT mice, whereas analysis of blood samples from the same mice bearing B16F10 cells for 6 h, 24 h, 48 h, and 72 h showed no significant differences in the number of B16F10 cells in the two groups (Fig. 3A). Quantitative analysis showed no significant differences in the percentage of B16F10 cells in the blood between WT and RNF13-KO mice (Fig. 3B). These results indicated that the deficiency of host RNF13 did not affect B16F10 cell clearance from the blood.

### Reduced GM-CSF concentration in the lungs of RNF13-KO mice

**Figure 4:**
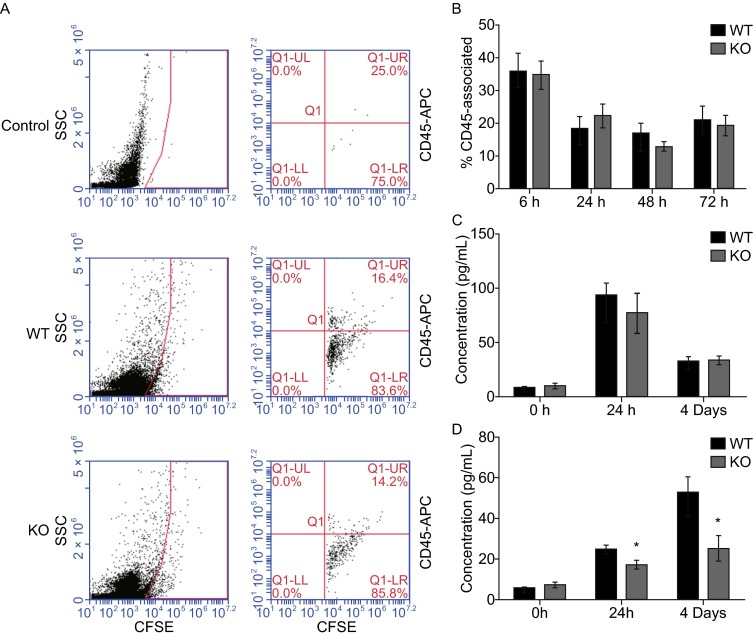
**Immune cells and cytokines detection in tumor bearing blood or lung tissues**. (A) At 24 h, blood was collected and analyzed for the presence of CFSE-B16F10. CFSE^+^ cells (gated as in left panels) were stained with CD45-APC (right panels). Note that CFSE^+^ cells are essentially absent in a sample from a control mouse with no B16F10 injection (top panel). (B) CFSE^+^ cells from the blood were analyzed to determine the percentage of CD45-engaged B16F10 cells at different time points (as in part A) (ns, 6 h *P* = 0.5349. *n* = 5, 24 h *P* = 0.4896. *n* = 6, 48 h *P* = 0.3812. *n* = 7, 72 h *P* = 0.7873. *n* = 5). (C) The concentration of GM-CSF in blood after bearing tumor for 24 h and 4 days in wild type and RNF13-KO groups. (ns, 0 h *P* = 0.6168. *n* = 5, 24 h *P* = 0.4982. *n* = 5,4 days *P* = 0.8897. *n* = 5). (D) GM-CSF was significantly reduced in RNF13-KO lungs compared with the wild type controls after bearing B16F10 cells (ns, 0 h *P* = 0.4223. *n* = 5; *, 24 h *P* = 0.0358. *n* =5, 4 days *P* = 0.0494. *n* = 5)

Immune cells and immune factors play a critical role in metastasis. Mechanisms that enhance the probability of CTCs successfully metastasizing to a distant organ mainly rely on physical interaction with leukocytes. Leukocytes in the blood support tumor cell colonization in metastatic organs (Man et al., 2011). However, the percentage of leukocytes (CD45-positive cells) was not increased in the blood of RNF13-KO mice at 6–72 h (Fig. S3). Furthermore, we hypothesized that a greater number of leukocytes interacting with B16F10 cells in RNF13-KO mice may enhance colonization in the lungs. To test this hypothesis, CFSE labeled B16F10 and CD45^+^ cells were detected in blood 24 h after injection of B16F10 cells into the blood of RNF13-KO and WT mice. Quantification of the number of CFSE and CD45 double positive cells indicated that the percentage of B16F10-engaged leukocytes did not differ significantly between RNF13-KO and WT mice (Fig. 4A and 4B). Similar results were obtained at 6 h, 48 h, and 72 h. The quantification of B16F10 relative leukocytes confirmed these findings (Fig. 4B). These results demonstrate that the lack of host RNF13 had no effect on the number of B16F10 relative leukocytes. Next, we evaluated the changes of cytokines in the blood and lungs from RNF13-KO and WT mice bearing B16F10 cells for 24 h and 4 days (PBS was used as control). Eighteen different cytokines or chemokines were detected by Luminex including GM-CSF, LIF, LIX, IL-1α, IL-1β, IL-4, IL-6, IL-10, IL-12 (P40), IL-12 (P70), IL-17, IFN-γ, TNFα, MIP-1α, MIP-1β, MIP-2, MCP-1, and RANTES. The concentration of these cytokines in the blood of RNF13-KO mice was comparable to that in WT mice (Fig. 4C). The results show that the concentration of GM-CSF in the lungs of RNF13-KO mice was lower than that in WT mice after bearing B16F10 cells (Fig. 4D).

### Enhanced invasion of B16F10 cells mediated by RNF13-KO conditioned media requires decreased GM-CSF

**Figure 5:**
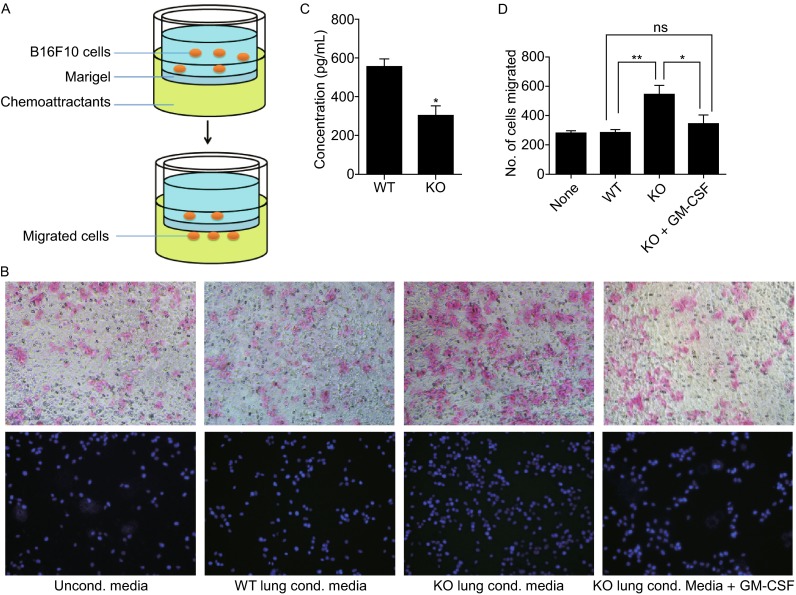
**Conditioned media from lung tissues of RNF13-KO mice promoted invasion of B16F10 cells in Transwell assays**. (A) Graphic representation of the methods used for determining B16F10 cell invasion in response to conditioned media. (B) The conditioned medium obtained from culturing WT lungs slices only modestly stimulated the invasion of B16F10 cells. The conditioned medium from RNF13-KO lungs caused a significantly higher level of cell invasion. Addition of GM-CSF to this medium rescued the increased migration B16F10 cells. Normal medium was used as a negative control in this experiment. (C) GM-CSF concentration in the conditioned media used in (B). Conditioned media from lung tissues of RNF13-KO mice was significantly reduced relative to that from WT mice (*, *P* = 0.0174). (D) The number of migrated cells was quantified (ns, *P* = 0.2785; *, *P* = 0.0478; **, *P* = 0.0014)

To determine whether the low concentration of GM-CSF in RNF13-KO lungs enhanced the invasive ability of B16F10 cells, we performed *in vitro* invasion assays (Fig. 5A). In these experiments, we used conditioned media generated by culturing slices of melanoma bearing lung tissues from either RNF13-KO mice or their WT littermates as chemoattractants. Assessment of the concentration of cytokines in conditioned media showed that, similar to the B16F10 bearing lungs, GM-CSF concentration was lower in conditioned media from RNF13-KO lungs (Fig. 5C). Transwell experiments showed that either nonconditioned media or the conditioned media generated by culturing WT lung slices only slightly stimulated the invasion of B16F10 cells from the upper to the lower chamber, whereas the conditioned media obtained from the culture of RNF13-KO lung slices dramatically stimulated the invasion of B16F10 melanoma cells (Fig. 5B and 5D). To determine whether this increased number of migrated cells was due to the reduction of GM-CSF concentration in RNF13-KO conditioned medium, recombinant GM-CSF was added to RNF13-KO conditioned media, which led to a reduction in the number of migrated cells (Fig. 5B and 5D). This means GM-CSF could successfully rescue the increased migrated cells caused by RNF13-KO conditioned media, and this rescue effect is in a dosage dependent manner (Fig. S4A and S4B). Taken together, our data indicate that host RNF13 may affect melanoma cell colonization in the lung by modulating the concentration of GM-CSF in the target organ, which may reduce the amount of B16F10 cells available for homing to the lung.

## DISCUSSION

RNF13 is an E3 ubiquitin ligase, and its association with pancreatic cancer was previously reported by our lab. RNF13 cannot be detected in pancreatic ductal epithelial cells, whereas it is expressed in pancreatic carcinoma precancerous lesion (chronic pancreatitis and pancreatic intraepithelial neoplasia) and pancreatic ductal adenocarcinoma (Zhang et al., 2009). These findings suggested that RNF13 may be involved in inflammation-associated carcinogenesis. To determine whether the lack of RNF13 affected the formation of spontaneous tumors and the survival rate, three different genotypes of mice (RNF13^+/+^, RNF13^+/−^, RNF13^−/−^) were observed for a period of 20 months or longer. Our results showed that the earliest time of death in RNF13-KO mice (12.2 months) and heterozygous mice (12.4 months) was earlier than that of WT mice (17.9 months), and their survival rate was lower (not significant) (Fig. S5). Analysis of the causes of death showed that the rate of cancer-related death was similar between RNF13-KO and WT mice. These results indicated that the lack of RNF13 did not affect the occurrence of cancer. However, our results did not exclude the possibility that RNF13 could function under pathological conditions by affecting the disease process, or its involvement in processes associated with cancer development.

In the present study, we confirmed that host RNF13 could block the colonization of metastatic tumor cells. Injection of B16F10 melanoma cells or LLC cells into the tail vein of mice enhanced pulmonary metastasis in RNF13-KO mice. In our previous research, RNF13 was downregulated during chicken skeletal muscle development, and RNF13 inhibited myoblast proliferation in chicken embryos (CFM) (Zhang et al., 2010). Therefore, we investigated whether deletion of host RNF13 may affect tumor cell proliferation and lead to enhanced metastasis. To test this hypothesis, we measured the size of metastatic foci in the lungs and found no significant differences between RNF13-KO and WT mice. Furthermore, we found that the lack of host RNF13 did not promote *in situ* LLC tumor growth.

It is reported that in animal studies, following intravenous injection, 60%–100% of the injected cancer cells are initially arrested in the lungs but after 24 h only 0.01% of circulating tumor cells (CTCs) could survive and eventually extravasate to form metastatic tumors (Fidler, 1970; Nicolson, 1979). Circulating tumor cells faced numerous challenges in blood before they could extravasate into distant organs. Mechanical destruction caused by the blood circulation and surveillance by immune cells contribute to making the blood clearance for a potential metastatic cell (Joyce and Pollard, 2009). In consideration of this issue, the differences of rapid clearance from blood could potentially result in more B16F10 cells being available for lung colonization in RNF13-KO mice. Therefore, we detect the percentage of B16F10 cells in blood in the short-term experiments (6–72 h after B16F10 injection). Although large discrepancy existed between individuals, the percentage of B16F10 cells in the blood was not increased in RNF13-KO mice. Moreover, further research using the lung tissues from same mice indicated an increased percentage of B16F10 cell available in the lung by flow cytometry. Furthermore, a greater number of B16F10 cells were detected in the lung tissues of RNF13-KO mice by frozen section at 6 h and 24 h or by H&E staining at day 2 and day 4. These results suggested that the loss of host RNF13 leading to increased B16F10 cell extravasation and homing.

We detected the concentration of 18 types of cytokines or chemokines in blood and tumor bearing lung tissues. The results showed that GM-CSF concentration was significantly decreased in RNF13-KO tumor bearing lungs. GM-CSF is an anti-tumor factor that is widely used as immune adjuvant in the treatment of many cancers including melanoma (Dranoff, 2002, 2003; Spitler et al., 2014). Tumor-derived GM-CSF is an important regulator of inflammation and immune suppression within the tumor microenvironment (Bayne et al., 2012). Increasing evidence suggests that GM-CSF enhances host responses through improved tumor antigen presentation by recruited immune cells, such as dendritic cells, leading to a reduction in metastasis (Williams et al., 2010). Eubank et al. (2009) found that GM-CSF can re-educate macrophages to reduce metastases in murine breast cancer. We therefore assumed that the low concentration of GM-CSF in RNF13-KO lungs could lead to an increase in metastatic B16F10 cells in the lung. To test this, we detected the concentration of cytokines in conditioned media from the culture of lung tissues, which showed that GM-CSF was lower in RNF13-KO than in WT mice media. Use of the conditioned media as a chemoattractant for *in vitro* invasion assays showed that the media from RNF13-KO cultures promoted B16F10 cell invasion. Rescue experiments further confirmed this result.

Our previous work showed that snapin is a substrate of RNF13. It was found that at hippocampal synapses snapin was ubiquitinated by RNF13 which promoted the association of snapin with SNAP-25 and regulated the function of SNARE complex (Zhang et al., 2013). According to the function role of SNARE complex in cytokine secretion, we considered that RNF13 may be involved in GM-CSF secretion through snapin (Stanley and Lacy, 2010). In this study we detected snapin expression between wild type and RNF13-KO mice after bearing B16F10 cells. The result indicated that snapin expression did not change in the RNF13-KO lungs (Fig. S6A and S6B). But whether RNF13 functions in metastasis are mediated through snapin or another substrate needs our further studies. Moreover, in order to find out whether the reduction of GM-CSF could lead to the expression changes of other genes which lead to the enhanced metastasis in RNF13-KO mice, we also detected expression patterns of several genes related to metastasis (Fig. S6C). But there are no significant differences between WT and RNF13-KO mice in these genes, including PECAM1, VCAM1, MAC-1, E-selectin, VE-cadherin, VEGF, and MMP9. Therefore, the exact mechanism of RNF13 function in metastasis needs to be systematically investigated in the future study.

In the present study, we showed that the deficiency of host RNF13 decreased the concentration of GM-CSF in the lungs, which facilitates metastatic cell colonization and leads to enhanced metastasis in RNF-13 KO mice. These findings may lead to the identification of novel therapeutic targets for the treatment of cancer metastasis.

## MATERIALS AND METHODS

### RNF13-KO mice

RNF13-KO mice were generated as described previously (Zhang et al., 2013) and housed under pathogen-free conditions at the Laboratory animal center of Peking Union Medical College. Genotype of all breeding pairs was confirmed by PCR analysis. In all studies, RNF13-KO mice, 7–12 weeks old, were compared with littermates of the same age and gender. Animal studies were conducted in accordance with the guidelines of the Animal Ethics Committee of Peking Union Medical College.

### Cells

The murine melanoma cell line B16F10 and Lewis lung carcinoma (LLC) cell line were obtained from American Type Culture Collection (Manassas, VA). Cancer cell lines were cultured in a humidified atmosphere of 5% CO_2_ using DMEM containing 10% fetal bovine serum, 100 U/mL penicillin G, and 0.1 mg/mL streptomycin. Tumor cell viability was greater than 95%, as determined by Trypan blue exclusion.

### *In vivo* tumor experiments

For assessment of local tumor growth, a suspension of 5 × 10^6^ LLC cells in PBS was injected subcutaneously into the dorsal region of RNF13-KO mice and their wild-type (WT) littermates. Starting at 8 days after implantation, when palpable tumors formed, tumor size was measured every 3 days and tumor volume was calculated using the formula length × width^2^ × 0.5. Mice were sacrificed 22 days after cell injection and tumor masses were collected and weighed.

For establishment of an experimental pulmonary tumor metastasis model, B16F10 melanoma cells were resuspended in PBS, and 5 × 10^5^ cells (in 0.2 mL PBS) were injected into mice via lateral tail vein. Mice were killed 14 days after injection and tissues were isolated and fixed in 10% neutral-buffered formalin. Lung lobes were photographed and metastatic foci on the surface of the lung were counted and their size was determined (using NIH Image J). For LLC tumors, the same procedure was used except that the cell number for tail vein injection was 1 × 10^5^ and mice were sacrificed 21 days after injection.

In the short-term metastasis model, B16F10 cells were labeled with CFSE or BCECF-AM (Molecular Probes, Invitrogen), and 5 × 10^5^ labeled B16F10 cells (in 0.2 mL PBS) were injected into the tail vein of mice. Control mice were injected with PBS. At 6 h after injection, mice were sacrificed and blood samples were collected directly from the heart into tubes containing 10 mmol/L EDTA in PBS to prevent coagulation. Erythrocytes were subsequently lysed followed by the detection of fluorescent B16F10 cells by flow cytometry. All five lobes of lungs were collected for later use in cryosections. Ten discontinuous sections were made for each lobe and more than 10 random fields were marked for each lobe; metastatic foci were visualized and counted using a fluorescence microscope. In some experiments, after blood collection, mice were perfused with 20 mL of PBS with 2 mmol/L EDTA until the lungs and liver appeared white. After perfusion, lungs were collected and processed by gentle mechanical disruption with forceps followed by enzymatic digestion with 2.4 U/mL dispase gradeII (Roche), 0.25 U/mL liberase TM Research Grade medium thermolysin (Roche), 20 Kunitz units/mL DNase (Sigma), and 20 U/mL hyaluronidase (Sigma), all dissolved in PBS. Tissue suspensions (5–6 mL per lung) were incubated at 37°C for 45 min with vigorous pipetting every 15 min to help tissue digestion. Cells were then strained through a 70 μm nylon mesh and suspended in PBS for detection by flow cytometry.

### Immune cell assay in blood

Blood and lung tissues were collected from RNF13-KO mice and their WT littermates bearing CFSE labeled B16F10 cells for 24 h, 48 h or 72 h. For blood assays, erythrocytes were subsequently lysed and for lung assays, tissues were perfused and digested as described above. Cells were then stained with fluorescently labeled monoclonal antibodies against CD45, washed in FACS buffer [PBS with 5% fetal bovine serum (FBS) and 2 mmol/L EDTA] and analyzed immediately by flow cytometry (using a BD Accuri™ C6 flow cytometer, BD Biosciences). Data were analyzed using CFlow® sampler analysis software (BD Biosciences).

### Cytokine detection

Lung tissues bearing B16F10 tumors for 24 h and 4 days were collected and digested with tissue lysis buffer (100 mmol/L TrisCl pH 8.0 buffer containing 0.1 mmol/L EDTA, 150 mmol/L NaCl, 1% NP-40, 20 mmol/L NaF, 1 mmol/L Na_3_VO_4_, 1 mmol/L NaH_2_PO_4_, and complete EDTA-free protease inhibitor cocktail). The samples were centrifuged to remove debris and assayed immediately. For blood detection, 24 h after the injection of B16F10 cells, blood was collected and clotted for at least 30 min before centrifugation to obtain serum for the next assay. Luminex assays (MILLIPLEX® MAP Kit Mouse cytokine/chemokine 96 well plate assay, Millipore) were performed for the detection of the following 18 cytokine or chemokine types: GM-CSF, LIF, LIX, IL-1α, IL-1β, IL-4, IL-6, IL-10, IL-12(P40), IL-12(P70), IL-17, IFN-γ, TNFα, MIP-1α, MIP-1β, MIP-2, MCP-1, and RANTES.

### Preparation of conditioned medium

B16F10 cells were injected into the tail vein of RNF13-KO mice and their WT littermates 24 h before perfused lungs were sectioned (2 mm^2^) and cultured in DMEM supplemented with 10% FBS for 90 min. The organ culture media were then centrifuged, and the supernatants were stored at −80°C until used as chemoattractants in the invasion assay.

### *In vitro* invasion assays

Twelve-well chambers fitted with 8.0 μm pore size polycarbonate filters (Corning) and Matrigel-coated (BD Biosciences) were used to perform the invasion assays. The lower compartment was filled with 1 mL of medium containing various chemoattractants. These chemoattractants included conditioned medium from the culture of B16F10-bearing WT and RNF13-KO lung sections and DMEM containing 10% FBS with recombinant GM-CSF (10 ng/mL); DMEM containing 10% FBS alone served as a control. B16F10 cells (5 × 10^4^) were resuspended in 500 µL of DMEM containing 0.1% bovine serum albumin and placed in the upper chamber. Cell invasion through the porous filter was allowed to take place for 24 h in a humidified atmosphere of 5% CO_2_ at 37°C. The upper surface of the filter was scraped with moist cotton swabs to remove nonmigrated cells, and then the cells attached to the lower surface of the filters were stained with eosin and DAPI. The invaded cells were photographed and counted in triplicate and at least 10 different fields for each repeat were analyzed.

### Statistical analysis

Data are expressed as the mean ± SE. The statistical significance of differences between two means was calculated using Student’s *t* test. *P* values < 0.05 were considered statistically significant.

## Electronic supplementary material

Supplementary material 1 (PDF 839 kb)
