# Supplementary material for: Enhanced metastasis in RNF13 knockout mice is mediated by a reduction in GM-CSF levels
Source: Protein Cell. 2015 Jul 22;6(10):746–56. doi: 10.1007/s13238-015-0188-7 (PMC4598322; doi:10.1007/s13238-015-0188-7)
Supplement: Supplementary file 1 — Supplementary material 1 (PDF 839 kb) [file 13238_2015_188_MOESM1_ESM.pdf]

Fig S1

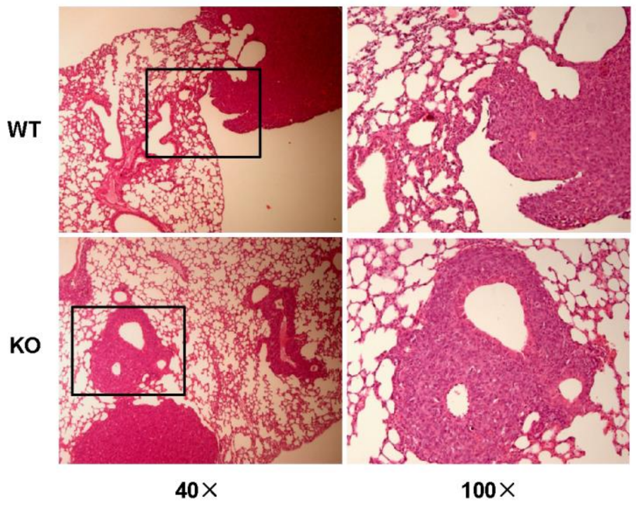

Fig. S1. Pathological section showing lungs in the LLC metastatic model. Representative H&E staining of sections of lung metastases from wild type and RNF13-KO mice. 40× magnification on the left panel and 100× magnification on the right panel.

Fig S2

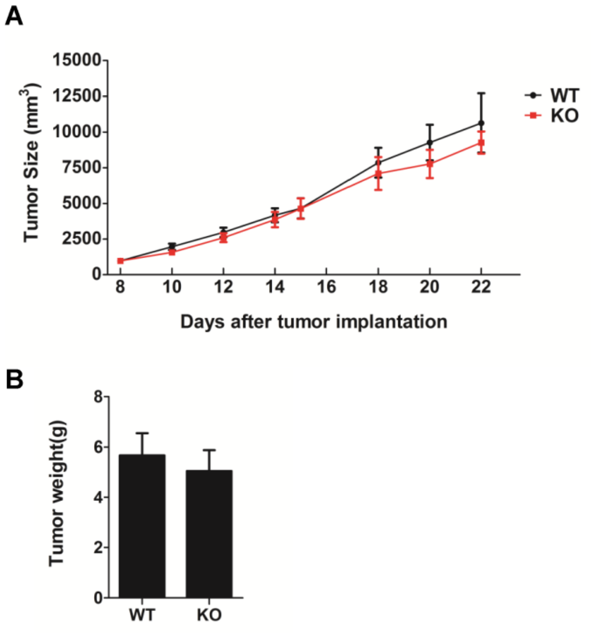

Fig. S2. Tumor growth was not correlated with loss of RNF13 function. (A) Growth curve of LLC subcutaneous tumors. (B) Tumor weight of RNF13-KO and wild type groups 22 days after bearing LLC cells (n=9).

Fig S3

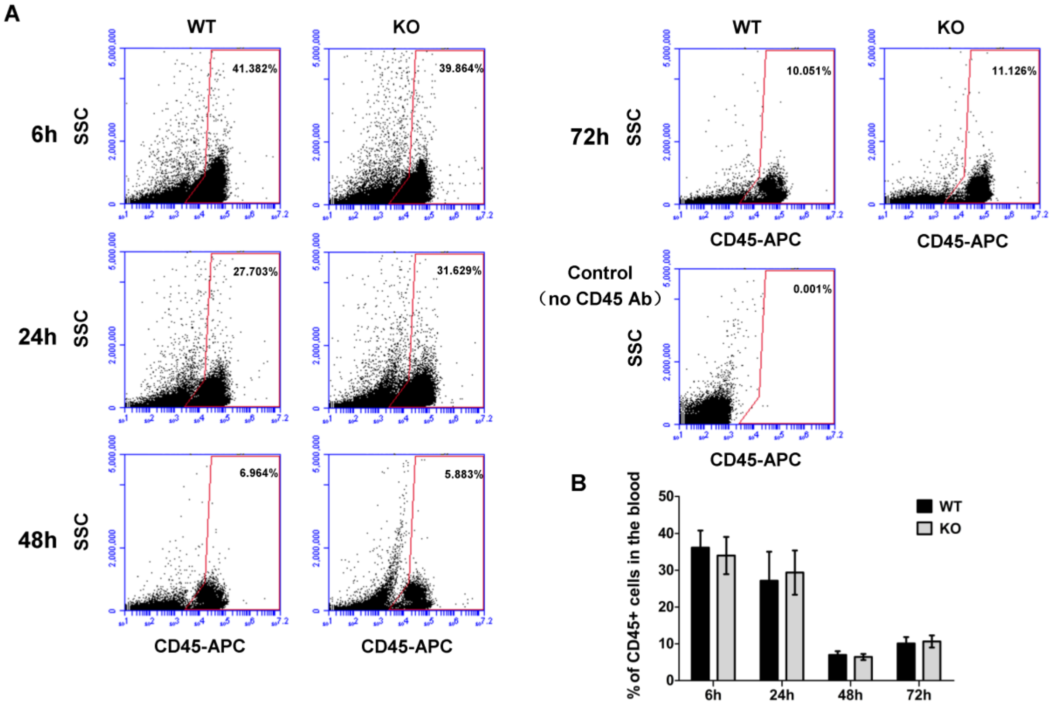

Fig. S3 The percentage of leukocytes was not increased in the blood of RNF13-KO mice. (A) Leukocytes (CD45-positive)in blood were detected by flow cytometry after bearing B16F10 cells for 6, 24, 48 or 72 hours. Note that CD45-APC cells are essentially absent in a sample without adding CD45-APC antidody (right panel). (B) Quantification showed the percentage of CD45+ cells in blood in wild type and RNF13-KO groups. (ns, 6h P=0.7569. n=5, 24h P=0.8253. n=6, 48h P=0.7003. n=7, 72h P=0.8249. n=5)

Fig S4

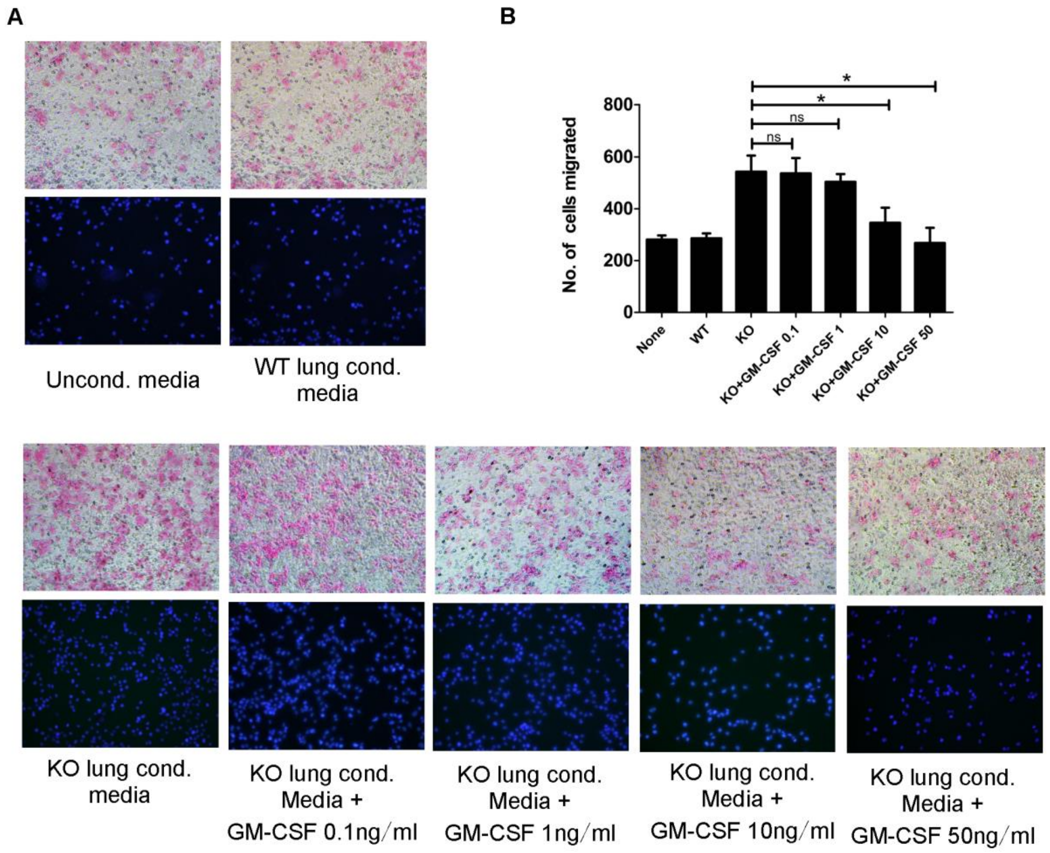

Fig. S4 GM-CSF dose-dependently rescued the enhanced invasion of B16F10 cells caused by RNF13-KO conditioned media in Transwell assay. (A) The ability of B16F10 cells to invasion was detected by Transwell assays. The lower compartment was filled with medium containing various chemoattractants. (B) Quantification showed the number of migrated B16F10 cells in each group. Addiction of GM-CSF could rescue the enhanced invasion in RNF13-KO group in a dosage dependent manner. (ns, "KO vs. KO+GM-CSF 0.1" P=0.9423 and " KO vs. KO+GM-CSF 1" P=0.5792; *, P=0.0478; **, P=0.0113)

Fig S5

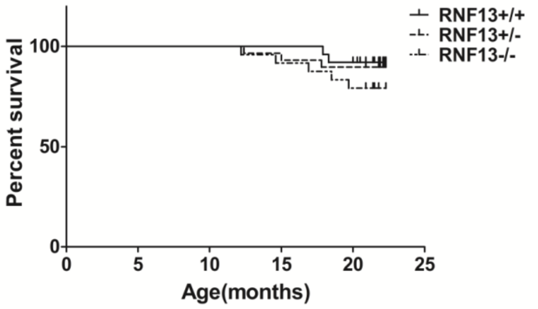

Fig. S5. Survival curves in mice with RNF13-KO (RNF13$^{-/-}$, n=24) heterozygous mice (RNF13$^{+/-}$, n=29) and in control wild type mice (RNF13$^{+/+}$, n= 25). The curves were developed using the Kaplan-Meier method and Log-rank Test (P=0.3562).

Fig S6

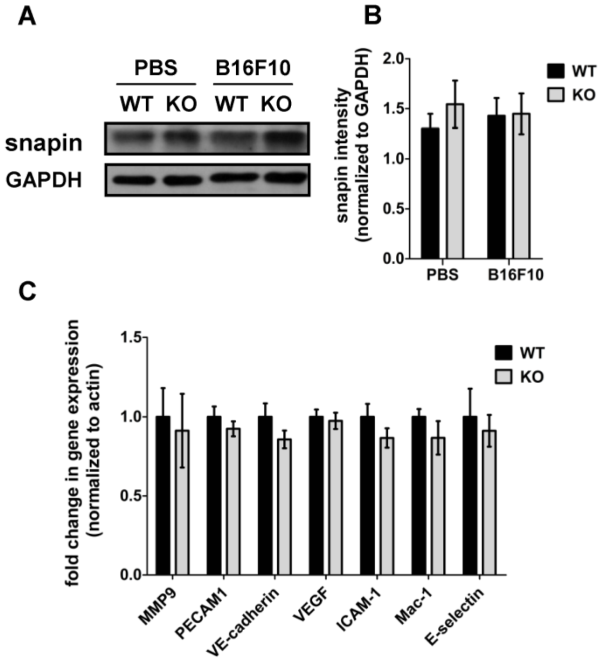

Fig. S6. Expression of metastasis related genes. (A) snapin expression in the lungs of WT and RNF13-KO mice bearing B16F10 cells or PBS as control.(B) Histograms showed the quantification in A (ns, WT-PBS to KO-PBS P=0.4328, WT-B16F10 to KO-B16F10 P=0.9428) . (C) The expression of some metastasis related gene in tumor bearing lungs were determined by qRT-PCR .(ns, MMP9 P=0.7860, PECAM1 P=0.4243, VE-cadherin P=0.2441, VEGF P=0.7432, ICAM-1 P=0.2747, Mac-1 P=0.3386, E-selectin P=0.7089.)
